# Staphylococcal persistence and biofilm resistance in bone-anchored hearing systems: Clinical impact

**DOI:** 10.1016/j.bioflm.2025.100342

**Published:** 2025-12-13

**Authors:** Marsel Ganeyev, Liliana Morales-Laverde, Maria Hoffman, Malou Hultcrantz, Anders Palmquist, Peter Thomsen, Martin L. Johansson, Margarita Trobos

**Affiliations:** a Department of Biomaterials, Institute of Clinical Sciences, Sahlgrenska Academy, University of Gothenburg, Gothenburg, Sweden; b Oticon Medical AB, Research & Technology, Askim, Sweden; c Centre for Antibiotic Resistance Research in Gothenburg (CARe), Gothenburg, Sweden; d Institution CLINTEC, Karolinska Institutet, Stockholm, Sweden; e Department of Biomedical Dental Sciences, College of Dentistry, Imam Abdulrahman Bin Faisal University, Dammam, Saudi Arabia

**Keywords:** Bone-anchored hearing systems (BAHS), *Staphyloccocus aureus*, *Staphyloccocus epidermidis,* biofilm formation, Antimicrobial resistance (AMR)

## Abstract

Persistent inflammation and infection, often linked to staphylococcal colonization, affect bone-anchored hearing system (BAHS) outcomes. Although antibiotics are often used to treat skin complications, the roles of biofilms and antimicrobial resistance (AMR) in clinical success remain unclear. This clinical prospective study characterized biofilm formation and antibiotic resistance in *Staphylococcus* spp. from BAHS patients, and examined associations with inflammation, pain, and hygiene. Adults eligible for BAHS were prospectively enrolled at a tertiary university hospital in Sweden during 2014–2015. Fifteen patients were followed clinically and microbiologically at surgery, 3- and 12- months. Abutment, peri-abutment exudate and soft-tissue samples were cultured. Fifty-seven *Staphylococcus* spp. isolates underwent biofilm phenotyping (Crystal Violet, Congo Red), antimicrobial susceptibility testing (minimum inhibitory concentration [MIC], minimum biofilm eradication concentration [MBEC]) and whole-genome sequencing (lineage, AMR and virulence genes). Clinical status was scored (Holgers, pain, debris). Individual patients harbored the same staphylococcal clone on abutment, exudate, and tissue for 12 months. *S. aureus* was more prevalent in patients with inflammation (Holgers score >0), *S. epidermidis* correlated with pain, and slime production was associated with debris accumulation. Overall, 56 % of isolates showed resistance to fusidic acid, and 11–34 % carried tetracycline resistance genes. *S. epidermidis* carried multidrug resistance genes (beta-lactams, tetracycline, sulfamethoxazole, fosfomycin), and resistance increased under biofilm conditions (MBEC > MIC). The *ica* operon was detected in all *S. aureus* and *S. epidermidis* ST7, ST297, ST749 and ST278. These findings indicate that staphylococci from BAHS exhibit persistent colonization, diverse clonal lineages, and high biofilm-associated AMR. Early microbial diagnostics and biofilm-targeted strategies, alongside cautious use of topical antibiotics, may improve outcomes.

## 1 Introduction

Bone-anchored hearing systems (BAHS) offer a reliable solution for patients with conductive or mixed hearing loss and unilateral deafness by bypassing the outer or middle ear and transmitting sound via a titanium fixture anchored in the temporal bone [1]. Despite their benefits, complications such as inflammation, infection, abutment loss, pain and numbness remain and can lead to surgical interventions or implant loss [2,3].

Calon TGA *et al*. (2019) described the microbiome associated with BAHS at 12 weeks and during inflammation [4]. *Staphylococcus epidermidis* and *Staphylococcus aureus* were found to be the most prevalent species in patients with and without signs of inflammation [[4], [5], [6], [7], [8]]. However, their specific roles in adverse tissue reactions are completely unknown, due to limited clinical studies and lack of methodological approaches.

Biofilm formation is a major virulence factor in staphylococcal species and plays a central role in biomaterial-associated infections. These biofilms, bacterial communities embedded in extracellular polymeric substances (EPS), protect bacteria from immune responses and antibiotics [9]. While a few studies confirm biofilm presence around bone-anchored implants [10], to our knowledge, none have examined how biofilm-associated bacterial properties relate to clinical outcomes in BAHS.

In BAHS practice, topical antibiotics are often used for prophylaxis or treatment of infection and pain. If ineffective, oral antibiotics may follow. Common treatments include ointments with (1) hydrocortisone acetate and oxytetracycline hydrochloride, polymyxin B sulfate [11,12], (2) fusidic acid [8] and others [13]. Silver nitrate cautery is occasionally used for treating granulation tissue [14]. However, antimicrobial resistance is rarely assessed in bacteria from BAHS, making treatment untargeted and potentially ineffective against biofilm-related resistance. This highlights the need to understand how bacterial colonization, biofilm formation and antibiotic resistance affect clinical outcomes in BAHS, especially infection and pain.

Previous studies have shown that abutment surface topography influences microbial colonization and inflammatory responses [8,[15], [16], [17]], with electropolished abutments linked to higher cytokine levels and bacterial loads, especially those of *S. epidermidis*. Combining MIC and MBEC assays provides a more complete view of resistance in biofilm-associated infections, especially in periprosthetic joint infections [18,19].

This study investigated the biofilm-forming capacity and antibiotic resistance of *Staphylococcus* spp. from BAHS patients. By correlating these virulence properties with adverse tissue reactions, we aimed to clarify their role in soft-tissue complications and inform improved infection management strategies.

## 2 Materials and methods

### 2.1 Study population, clinical parameters, and outcomes

#### 2.1.1 Study design

Adult patients, eligible for BAHS, were enrolled and the surgery was performed in a tertiary university hospital in Sweden between November 2014 and December 2015. Sixteen adults received BAHS, one was excluded due to early implant loss, leaving 15 patients for analysis. Clinical and microbiological assessments were conducted at baseline, 3, and 12 months postoperatively. All patients received Wide Ponto 4 mm implants (Oticon Medical AB, Sweden) with 6-, 9- or 12-mm abutments, installed via the Minimally Invasive Ponto Surgery (MIPS) technique [8]. Demographics, hearing loss indications, and abutment lengths are shown in Table 1.

**Table 1:** Patient demographics and abutment lengths.

Baseline demographics	Overall	Male	Female	
*Patients n (%)*	15 (100)	7 (47)	8 (53)	
*Age mean years (SD)*	54.3 (24.7)	61.7 (24.7)	47.8 (24.5)	
*Type of hearing loss n (%)*				
Acquired	7 (46)	3 (42)	4 (50)	
Congenital	4 (27)	2 (29)	2 (25)	
Single-sided	4 (27)	2 (29)	2 (25)	
*Smokers n (%)*				
No Smoking	15 (100)	7 (100)	8 (100)	
Smoking	0	0	0	
*Body mass index mean (SD)*	27.20 (7.4)	25.21 (4.7)	29.87 (8.9)	
*Abutment length n (%)*				
Abutment 6 mm	1 (7)	0	1 (12)	
Abutment 9 mm	9 (60)	6 (86)	3 (38)	
Abutment 12 mm	5 (33)	1 (14)	4 (50)	

#### 2.1.2 Sampling and examination

Sampling was previously described by Trobos *et al*. [8]. Quantitative bacterial cultures of abutment, periabutment fluid, and soft tissue biopsies were performed within 2 days of retrieval (Fig. S1 in **Supplement 1**). From staphylococci-selective media, one colony per patient/sample/time-point was subcultured and stored for analysis.

Holgers scores [0 (no irritation) – 4 (abutment removed due to persistent infection)] [20], pain scores [0 (no pain) – 10 (worst possible pain)], and debris scores [0 (no debris) – 3 (abundant debris around abutment)] were recorded. After implantation, dressings soaked in ointment containing oxytetracycline hydrochloride (0.5 % w/w) and polymyxin B sulfate (10,000 IU) were applied around the abutment for up to 7 days. Adverse tissue reactions were treated with the same ointment and/or lapis (75 %). Two patients received fusidic acid (2 % w/w) ointment up to 3 × daily for 2 weeks.

Clinical, microbiological, and molecular outcomes were previously reported [8,15]. Staphylococcal strains were stored at −80 °C in a biobank. This report evaluates their phenotypic/genotypic virulence and clinical associations.

## 3 Phenotypic and genotypic properties of the strains

### 3.1 Biofilm formation: Crystal Violet and Congo Red

The biofilm-forming ability of the strains was assessed. Biofilm biomass was quantified using the Crystal Violet (CV) microtiter plate test. Overnight cultures in tryptic soy broth (TSB) with 0.25 % glucose (for *S. aureus*) were adjusted to OD_546_ = 1, diluted 1:40, and inoculated (200 μL, triplicate) into 96-well plates (Thermo Scientific™, USA). After 24 h at 37 °C, wells were washed, stained with 2 % CV, destained with ethanol-acetone (80:20), and blank-subtracted OD_595 nm_ values were used to classify strains according to reported thresholds [21].

Slime production was assessed using Congo Red (CR) agar [22]. Strains were plated on brain heart infusion agar supplemented with sucrose and CR, incubated at 37 °C for 24–48 h, and classified by colony color: very red (vr), red (r), and bordeaux (brd) corresponded to non-slime producers (NP); almost black (ab) indicated weak slime production (WP); and black (b) to very black (vb) represented slime producers (P).

Reference strains included *S. epidermidis* ATCC 35984/12228 and *S. aureus* 15981 wild-type/Δ*ica.*

### 3.2 Antimicrobial susceptibility: MIC and MBEC

Minimum inhibitory concentrations (MICs) were determined via microbroth dilution following EUCAST guidelines [23] using a custom-made antimicrobial plate (Sensititre™ SWE1GOTH, Thermo Fisher Scientific™, UK) [24] (Fig. S2 in **Supplement 1)**. Inoculums (5 × 10^5^ CFU/mL per well) in Mueller-Hinton Broth 2 cation-adjusted (MHB, Sigma Aldrich, USA) were incubated at 37 °C for 20 h. MICs were visually assessed using the Sensititre™ Viewbox, with *S. aureus* ATCC 29213 as control. Strains were categorized as Susceptible (S), Intermediate (I), or Resistant (R) according to EUCAST breakpoints [23].

Minimum biofilm eradication concentrations (MBECs) for 9 antimicrobial agents were assessed in slime-producing strains, as previously described [25]. The Calgary Biofilm Device (MBEC Assay®, Innovotech Inc., Canada) was inoculated with 5 × 10^5^ CFU/mL of each strain in MHB and biofilms were grown for 24 h on peg lids, then exposed to antibiotics for 20 h at 37 °C. Then, pegs were sonicated in neutralizing medium and incubated overnight. Finally, MBECs were determined visually and compared to resistance thresholds. If growth persisted at the highest concentration, the next doubling concentration was used for MBEC/MIC ratios.

### 3.3 Bacterial typing and virulence genes

Genomic DNA was extracted using the GenElute Bacterial Genomic DNA Kit (Sigma-Aldrich, USA) with Gram-positive protocol, lysozyme, lysostaphin, and RNase A treatments. Libraries were prepared using Nextera XT (Illumina, USA) and sequenced on MiSeq (Illumina) (≥40 × coverage). Bioinformatics (1928 Diagnostics, Sweden; Center for Genomic Epidemiology, DTU, Denmark) included core-genome phylogeny, species identification, multilocus sequence typing (MLST), and virulence gene screening. *agr* types and *ica* operon genes were identified using BLASTn (≥90 % identity). Raw data are available in the European Nucleotide Archive (ENA) (BioProject PRJEB89840). The detailed protocol is available in S**Methods**, **Supplement 2**.

## 4 Statistical analysis

Data are presented as mean ± SD. Chi–square test and Fischer's exact test assessed associations betweenspecies, biofilm, and tissue reactions. Univariate logistic regression evaluated associations between biofilm biomass/slime and clinical outcomes. One-sample t-tests compared intra-species CV OD values; independent t-tests and one-way ANOVA assessed differences in CV OD and MBEC/MIC ratios. Significance: *p* < 0.05. Analyses were performed using GraphPad Prism (GraphPad Software, USA).

## 5 Results

### 5.1 Study participants and clinical outcome

A total of 57 staphylococcal strains were isolated from soft tissue, peri-abutment fluid, and abutments of 15 patients:16 *S. aureus*, 32 *S. epidermidis,* and 9 other coagulase-negative staphylococci (CoNS) (3 *S. lugdunensis*, 3 *S. schleiferi*, 1 *S. capitis* and 2 *S. hominis*). Isolates were collected at baseline (n = 1), 3 months (n = 33), and 12 months (n = 21). Two additional strains were isolated at 1 month from a patient with implant loosening.

Seven patients exhibited minimal or moderate debris; four had Holgers score >0, indicating skin inflammation, and six reported pain at least once.

*S. epidermidis* was identified in 13 out of 15 patients, whereas *S. aureus* in five, and other CoNS in six. The presence of *S. aureus* was significantly associated with Holgers score >0 (*p* = 0.0148) (Table 2). Pain was most frequently reported in patients colonized by *S. epidermidis* (76 %, 13/17; *p* = 0.0537).

**Table 2:** Correlation between clinical outcome and the species and biofilm formation the colonizing strain.

	Biofilm formation (CV)		Biofilm slime production (CR)	
**Bacterial species**	NP and WP, n = 19	MP and SP, n = 38	Non-slime producers, n = 20	Slime producers, n = 37
*S. aureus,* n = 16 (100 %)	2 (12)	14 (88)	0	16 (100)
*S. epidermidis,* n = 32 (100 %)	12 (37)	20 (63)	14 (44)	18 (56)
Other CoNS, n = 9 (100 %)	5 (56)	4 (44)	6 (67)	3 (33)
*p*-value	0.0736		0.0002∗∗∗	

Bacterial species	Clinical outcomes/Adverse reactions				
	Holgers>0, n = 16 (100 %)	Pain, n = 17 (100 %)	Debris, n = 25 (100 %)	No adverse reactions, n = 25 (100 %)	Any adverse reaction, n = 32 (100 %)
*S. aureus*, n = 16	9 (56)	4 (24)	10 (40)	6 (24)	10 (31)
*S. epidermidis*, n = 32	6 (38)	13 (76)	11 (44)	14 (56)	18 (56)
Other CoNS, n = 9	1 (6)	0	4 (16)	5 (20)	4 (13)
*p-*value	0.0148∗	0.0537	0.1713	0.2307	
**Biofilm formation (CV)**					
Non- and weak- producers, n = 19	6 (38)	9 (53)	8 (32)	7 (28)	12 (38)
Moderate- and strong-producers, n = 38	10 (62)	8 (47)	17 (68)	18 (72)	20 (62)
*p-*value	0.7583	0.0646	0.8503	0.4503	
**Biofilm slime production (CR)**					
Non-slime producers, n = 20	3 (19)	7 (41)	4 (16)	11 (44)	9 (28)
Slime producers, n = 37	13 (81)	10 (59)	21 (84)	14 (56)	23 (72)
*p-*value	0.1323	0.5570	0.0114∗	0.2682	

Note: The classification of biofilm production follows the criteria established by Baldassarri *et al*. [21], where non-producers (NP) correspond with OD_570_ < 0.120, weak producers (WP) OD_570_ = 0.120 to 0.240, moderate producers (MP) OD_570_ = 0.240 to 0.480, and strong producers (SP) OD_570_ > 0.480. Statistics: Pearson's Chi–Square test and Fischer's exact test were used for the comparisons of categorical variables with *p* ≤ 0.05 considered significant. Abbreviations: CoNS = coagulase-negative staphylococci; CV = Crystal Violet; CR = Congo Red.

### 5.2 Phylogenetic evaluation of the entire strain population

**Fig. 1:**
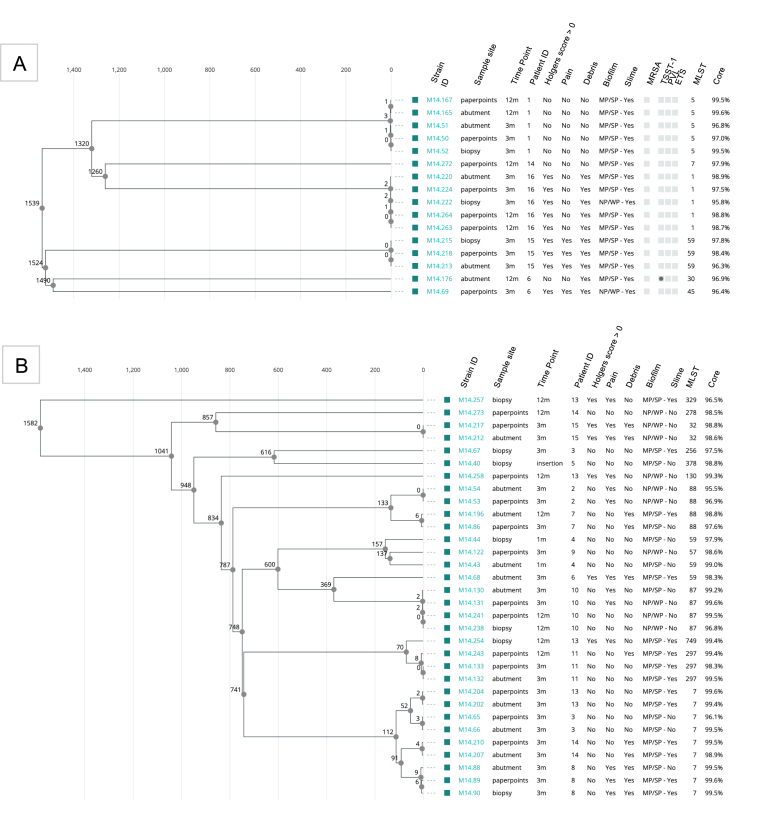
Phylogenetic tree. Core-genome multilocus sequence typing (cgMLST)-based phylogenetic trees of *Staphylococcus aureus* (A) and *Staphylococcus epidermidis* (B) strains from BAHS patients. Trees illustrate allelic distances and clustering of strains based on sequence types (STs), highlighting potential clonal dissemination and associations with biofilm formation, antibiotic resistance profiles, and clinical outcomes (Holgers score >0, pain, debris).

Phylogenetic relationships among 16 *S. aureus* and 32 *S. epidermidis* strains were visualized in a cgMLST-based tree (Fig. 1). Most strains from the same patient were closely related, regardless of time or site of isolation. Six sequence types (STs) were identified in *S. aureus*, with ST5 and ST1 being most common and each linked to one patient. Twelve STs were identified in *S. epidermidis*, with ST7 most prevalent (33 %, 10/32) and found in four patients.

### 5.3 Biofilm forming ability

Biofilm biomass was significantly associated with slime production (OR = 11.56, *p* = 0.0002) (Fig. S3A in **Supplement 1**). The model showed good discrimination (AUC = 0.76) and explained 28 % of the variance (Tjur's R^2^ = 0.28). CV identified 19 strains (33 %) as non/weak biofilm producers (NP/WP) and 38 (67 %) as moderate/strong biofilm producers (MP/SP) (Table 2). CR identified 20 non-slime producers (35 %), 12 weak slime producers (21 %), and 25 strong slime producers (44 %). Weak and strong slime producers were grouped as “slime producers” for analysis.

Slime production varied significantly by species (*p* = 0.0002) (Table 2). Most *S. aureus* strains were biofilm biomass (CV: 88 %) and slime (CR: 100 %) producers (Table 2). All carried the *ica* operon, which is responsible for PIA synthesis (polysaccharide intercellular adhesin). *S. epidermidis* exhibited similar biofilm biomass (CV: 63 %) and slime (CR: 56 %) production (Table 2); 14/32 carried the *ica* operon (Table 3). Slime production in *S. epidermidis* was strongly associated with *ica* carriage, especially in ST7 and ST297. Other CoNS were mostly non-producers (67 %) (Table 2).

**Table 3:** MLST-based clonal characterization, antimicrobial resistance, and virulence profiles.

*Staphylococcus aureus*							
MLST	Spa type	Phenotypic AMR	AMR genes	Agr type	*ica*	Slime	Virulence genes
ST 5 (n = 5)	t2			2	Yes	Yes	*aur, hlgA, hlgB, hlgC, lukD, lukE, seg, sei, sem, sen, seo, sep, seu, sak, scn, splA, splB*
ST 1 (n = 5)	t127	FUS	*blaZ, tet(K) (n = 1)* *blaZ, fusC (n = 2)* *blaZ, fusC, tet(K) (n = 1)*	1	Yes	Yes	*aur, hlgA, hlgB, hlgC, lukD, lukE, sea, seh, sek, seq, sak, scn, splA, splB, splE*
ST 59 (n = 3)	t437			1	Yes	Yes	*aur, hlgA, hlgB, hlgC, seb, sek, seq, scn*
ST 30 (n = 1)	t338		*blaZ, erm(A), ant(9)la*	3	Yes	Yes	*aur, hlgA, hlgB, hlgC, lukD, lukE, sea, seg, sei, sem, sen, seo, seu, sak, scn, tst*
ST 45 (n = 1)	t230		*tet(K)*	1	Yes	Yes	*aur, hlgA, hlgB, hlgC, lukD, lukE, sec,sec3, seg, sei, sel, sem, sen, seo, seu, sak, scn*
ST 7 (n = 1)	t91			1	Yes	Yes	*aur, hlgA, hlgB, hlgC, sea, seg, sei, sem, sen, seo, seu, sak, scn, splE, tst*

*Staphylococcus epidermidis*					
**MLST**	**Phenotypic AMR**	**AMR genes**	**Agr type**	***ica***	**Slime**
ST 7 (n = 10)	FUS (n = 4) SXT (n = 3) CLI, FUS (n = 1)	*blaI/blaR1, fusB, dfrS1, fosB, msr(A); mph(C), tet(K), (n = 1)* *blaZ/blaI/blaRI, fusB, dfrS1, fosB, msr(A); mph(C), tet(K), (n = 1)* *blaZ/blaI/blaRI, fusB, dfrS1, fosB, msr(A); mph(C), (n = 2)* *blaZ/blaI/blaRI, dfrS1/dfrG, fosB, msr(A); mph(C), tet(K), (n = 5)* *blaZ/blaI/blaRI, vga(A), fusB, dfrS1, fosB, msr(A); mph(C), (n = 1)*	1 (n = 6) 3 (n = 1)	Yes (n = 9) No (n = 1)	Yes
ST 88 (n = 4)	FUS (n = 2) OXA, FOX, FUS (n = 1) OXA, FOX, FUS, SXT (n = 1)	*blaZ/blaI, fusB, dfrS1, fosB, (n = 1)* *blaZ, fusB, dfrS1, fosB, (n = 1)* *mecA/blaZ/blaI/blaRI, mecA, fusB, dfrS1, fosB, tet(K), (n = 2)*	3 (n = 4)	No	No (n = 3) Yes (n = 1)
ST 87 (n = 4)	FUS (n = 4)	*blaZ/blaI, fusB, dfrS1, aadD/ant(9)-Ia, fosB, msr(A); mph(C), (n = 4)*	1 (n = 1)	No	No
ST 59 (n = 3)	FUS (n = 2)	*blaZ/blaI/blaR1, fusB, dfrS1, fosB, (n = 1)* *dfrS1, fosB, msr(A); mph(C), tet(K), (n = 1)* *blaZ/blaI, fusB, dfrS1, fosB, (n = 1)*	1 (n = 2) 2 (n = 1)	No	No (n = 2) Yes (n = 1)
ST 297 (n = 3)	FUS (n = 3)	*fusB, dfrS1, fosB, msr(A); mph(C), (n = 3)*	1	Yes	Yes
ST 32 (n = 2)	FUS (n = 2)	*blaZ/blaI/blaRI, fusB, dfrS1, fosB, (n = 2)fusB, dfrS1, fosB, msr(A); mph(C), (n = 1)*	2	No	No
ST 749 (n = 1)		*dfrS1, fosB*	1	Yes	Yes
ST 130 (n = 1)	FUS	*fusB, dfrS1, fosB*	1	No	No
ST 278 (n = 1)	FUS	*blaZ, fusB, dfrS1, fosB, msr(A); mph(C)*	3	Yes	Yes
ST 57 (n = 1)			1	No	No
ST 378 (n = 1)		*blaZ/blaI/blaR1, dfrS1, fosB*	3	No	No
ST 256 (n = 1)		*dfrS1, fosB, tet(K)*	1	No	Yes

Among strains linked to tissue reactions and/or pain, 63 % (20/32) were biofilm producers and 72 % (23/32) slime producers (Table 2). Slime producers were significantly associated with visible debris around the abutment (*p* = 0.0114). Patients with high Holgers scores were mainly colonized by biofilm (62 %) and slime (81 %) producers. No correlation was found between biofilm production and pain.

Heat maps (Fig. S3B and C) showed *S. aureus* was prevalent but less common in patients experiencing multiple adverse reactions or pain during follow-up. In contrast, *S. epidermidis* was more frequent in patients with 0–1 episodes of adverse reactions. Slime producers and non-producers were frequently linked to patients with or without episodes of adverse tissue reactions or pain.

### 5.4 Antimicrobial susceptibility under planktonic (MIC) and biofilm (MBEC) conditions

**Fig. 2:**
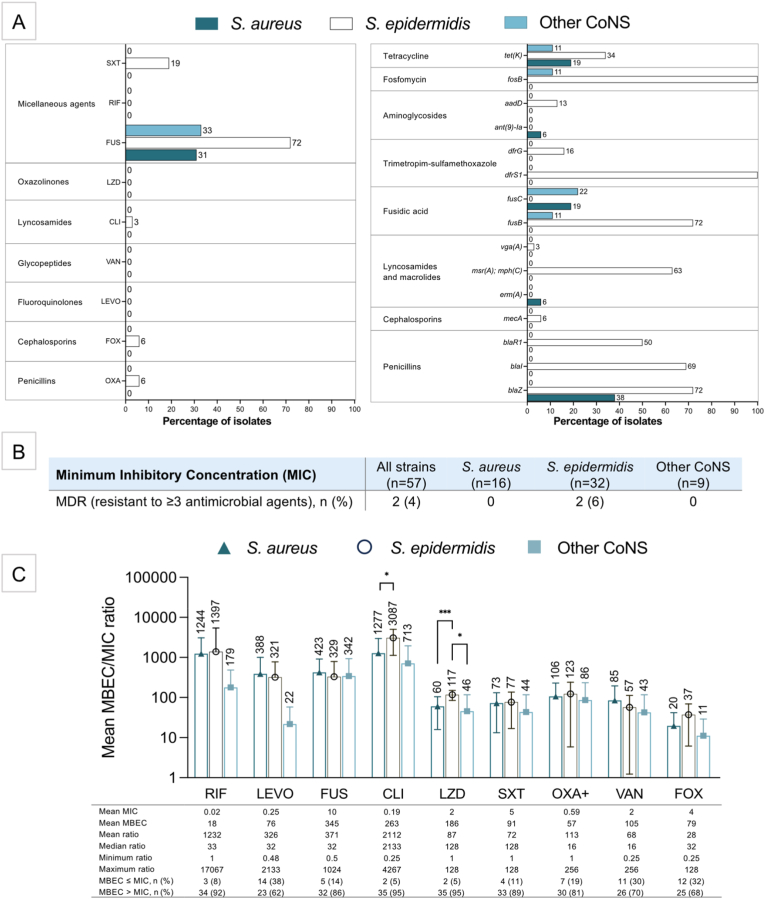
Phenotypic and genotypic antimicrobial resistance profiles of staphylococci isolated from BAHS. (A) Prevalence of antimicrobial resistant strains (according to MIC) and resistance gene profiles. EUCAST breakpoints were used. (B) Prevalence of multidrug resistance (MDR). (C) Mean MBEC/MIC ratios categorized by bacterial species indicating that staphylococcal strains from BAHS exhibit increased antibiotic resistance when grown as biofilms. Abbreviations: minimum inhibitory concentration (MIC), minimum biofilm eradication concentration (MBEC), Rifampicin (RIF), Levofloxacin (LEV), Fusidic Acid (FUS), Clindamycin (CLI), Linezolid (LZD), Trimethoprim/Sulfamethoxazole (SXT), Oxacillin (OXA+), Vancomycin (VAN), Cefoxitin (FOX).

All 57 bacterial strains were tested for MIC; 37 slime-producing strains were also tested for MBEC. All strains were susceptible (S) to RIF, LZD, VAN and LEVO, while 54 % were resistant (R) to FUS (33 % *S. aureus*, 72 % *S. epidermidis*, 31 % other CoNS) (Fig. 2A; STable 1 in **Supplement 1**). FUS resistance genes (*fusC and fusB*) were frequently detected in *S. aureus* (19 %), *S. epidermidis* (72 %), and other CoNS (22 and 11 %). The tetracycline resistance gene *tet(K)* was also common: *S. aureus* (19 %), *S. epidermidis* (34 %), and other CoNS (11 %).

In *S. epidermidis*, resistance was observed for SXT (19 %), CLI (3 %), FOX (6 %), and OXA+ (6 %) with the highest prevalence of resistance genes (Fig. 2A). All carried genes for fosfomycin (*fosB*) and sulfamethoxazole resistance (*dfrS1*) (16 % also had *dfrG)*, and many had genes for β-lactam (*blaZ*, *blaI*, *blaR1)*, macrolide (*mphC*, and *msrA, vga(A)*), and tetracycline (*tet(K)*) resistance. One ST88 clone carried *mecA* and *aadD/ant(9)-Ia*, conferring methicillin and aminoglycoside resistance, respectively, and was multidrug resistant (MDR) to ≥ 3 antibiotics (Fig. 2B).

Slime-producing strains showed increased resistance in MBEC assays (STable 1). RIF, LEVO, LZD and VAN, effective against planktonic bacteria (MIC), showed 68–92 % resistance in MBEC. FUS resistance increased from 54 % (MIC) to 97 % (MBEC), and SXT resistance from 11 % (MIC) to 78 % (MBEC).

Mean MBEC/MIC ratios illustrate the fold increase in AMR of biofilms and facilitate comparisons between species (Fig. 2C), ranging from 28 (FOX) to 2112 (CLI). *S. epidermidis* exhibited significantly higher MBEC/MIC ratios for CLI than *S. aureus* (*p =* 0.0165) and for LZD (vs. *S. aureus*, *p* = 0.0008; vs. other CoNS, *p* = 0.0229), indicating stronger biofilm tolerance.

### 5.5 Carriage of virulence factors

*S. aureus* strains exhibited diverse genotypic and phenotypic profiles (Table 3). Multiple clones, such as ST5 and ST1, carried virulence genes associated with enhanced pathogenicity, such as staphylococcal enterotoxins (*sea, sec, seg*), leukocidins (*lukD* and *lukE*), and superantigens (*sei*, *sem*, *sen*). Immune evasion genes included *scn* (staphylococcal complement inhibitor) and *sak* (staphylokinase). ST30 and ST7 carried *tst* (toxic shock syndrome toxin). All *S. aureus* strains were slime producers and carried the *ica* operon. ST1 isolates were FUS-resistant and carried *fusC*.

*S. epidermidis* strains were genetically diverse, with common sequence types ST7, ST88, and ST87. Most*,* except ST57, carried multiple resistance genes such as *blaI/blaR1*, *fusB*, *dfrS1*, *fosB*, *msrA*, *mphC*, *tetK*, and *mecA* (notably in ST88). Phenotypic resistance generally matched genotypic profiles; (e.g., FUS resistance was linked to *fusB*). Some strains (ST7, ST297, ST278) carried *ica* and produced slime, while others (ST88, ST256) produced biofilms independently of *ica*.

## 6 Discussion

### 6.1 Clonal persistence and epidemiology

Core genome MLST demonstrated that patients can remain colonized by the same clone across multiple sites and time points, up to 12 months post-implantation. In this study, the term “persistence” refers to sustained colonization or clonal presence of staphylococci within individual hosts over time, as evidenced by repeated isolate recovery and genomic analysis. This includes clonal persistence across multiple sites and time points and species-specific associations with clinical outcomes. This suggests persistent colonization or low-grade infection by specific clones within individual hosts, highlighting the challenge of eliminating these bacteria from patients with BAHS. Such long-term colonization may be associated with skin-associated adverse events or increased risk of infections.

While *S. aureus* sequence types (STs) were patient-specific, *S. epidermidis* showed broader dissemination (twelve STs). ST7 was the most prevalent in *S. epidermidis* from BAHS and has been previously associated with catheter-related bloodstream infections and prosthetic valve endocarditis, indicating its potential for implant-associated pathogenicity [[26], [27], [28]]. Interestingly, high-risk clones such as ST2, previously reported in PJI to combine multidrug resistance with strong biofilm formation, were not detected, suggesting a diverse but less virulent *S. epidermidis* population in this cohort [19,29]. *S. epidermidis* was more resistant than *S. aureus*, often harboring multiple antimicrobial resistant genes. This suggests potential for multidrug-resistance and increased treatment failure, consistent with findings from other clinical settings [19]. When comparing BAHS-associated staphylococcal isolates to those reported in periprosthetic joint infections (PJIs), some similarities and notable differences emerge. For *S. aureus*, BAHS isolates share common sequence types with lineages previously identified in PJIs, such as ST45, ST30, and ST5, suggesting that BAHS complications may exhibit epidemiological similarities to other device-associated staphylococcal infections [19]. In a previous study, *S. epidermidis* PJI isolates were dominated by high-risk clones such as ST2 and ST5, which were associated with multidrug resistance (MDR) and strong biofilm formation, with MDR rates reported at 78 % [19]. In contrast, BAHS *S. epidermidis* isolates exhibited greater clonal diversity (e.g., ST7, ST88, ST297) and a markedly lower MDR prevalence (6 %). The increased MDR observed in PJIs could reflect the prolonged antimicrobial therapy typically required for these infections, with treatment durations averaging 15.6 weeks (intravenous and oral), which may exert strong selective pressure for resistant strains [18,19].

### 6.2 Species-specific clinical associations

*S. aureus* colonization was significantly associated with increased Holgers scores, indicating a stronger link to skin inflammation [30]. This aligns with its known virulence, including the presence of *scn* and *sak* (linked to human-specific innate immune modulation) [31], *lukD/E*, and enterotoxin genes, and its ability to invade keratinocytes [32], which may contribute to its persistence within the host [31]. In contrast, *S. epidermidis* was more frequently associated with reported pain, consistent with its role in subacute or chronic infections [33]. Similar trends were observed in patients with orthopedic implants, where staphylococci were strongly associated with persistent infections and poor clinical outcomes [19,[34], [35], [36]]. Despite being a skin commensal, *S. epidermidis* is a leading cause of prosthetic joint infections [35] due to its ability to form biofilms, making it an opportunistic pathogen. *S. epidermidis* may also contribute to the development of common skin diseases [29] by producing cysteine proteases. These enzymes can exacerbate skin inflammation [37], highlighting their potential contribution to skin-associated adverse reactions.

### 6.3 Biofilm formation and clinical impact

Biofilm formation was assessed at a standardized 24 h time point to compare the biofilm forming abilities of the isolates under controlled *in vitro* conditions. Biofilm formation was widespread in *S. aureus* (CV: 88 %, CR: 100 %) and *S. epidermidis* (CV: 63 %, CR: 56 %). The *ica* operon, essential for PIA synthesis, was present in all *S. aureus* and 44 % of the *S. epidermidis* (ST7, ST297, and ST278). Some *S. epidermidis* clones (ST88 and ST256) produced biofilms independently of *ica*, suggesting alternative biofilm mechanisms.

When compared with previously reported PJI isolates, BAHS-associated staphylococci displayed similar biofilm-forming abilities. In PJI cohorts, moderate-to-strong biofilm formation was observed in 82 % of *S. aureus* and 66 % of *S. epidermidis* isolates [19], whereas in our BAHS cohort the proportions were 88 % and 63 %, respectively. These comparable levels may suggest that BAHS-associated strains retain a biofilm-forming phenotype characteristic of implant-associated staphylococci, despite their distinct ecological niche and reduced multidrug resistance. This similarity also underscores the clinical relevance of biofilm formation in BAHS complications and supports parallels with other device-related infections.

Slime-producing strains were significantly associated with visible debris around the abutment, and most strains from patients with elevated Holgers scores or debris were biofilm producers. These findings support the hypothesis that biofilm formation contributes to soft-tissue complications in BAHS, as previously observed in other percutaneous implants [24].

### 6.4 Antimicrobial resistance and clinical implications

A major concern is the high prevalence of fusidic acid resistance (FUS^R^) (54 % overall; 72 % in *S. epidermidis*), likely driven by the routine aftercare use of fusidic acid ointments. Antimicrobial resistance (AMR) genes (*fusB*, *fusC*) were frequently detected, and FUS^R^ increased dramatically under biofilm conditions (MBEC: 97 %). Varying FUS^R^ rates in *S. aureus* (0.3–52.5 %) [[38], [39], [40], [41], [42]] and *S. epidermidis* (7.7–46 %) [43,44] have been reported in skin, respiratory, and bone infections across countries. In a UK dermatology clinic, high FUS^R^ in *S. aureus* prompted efforts to reduce fusidic acid ointment use [45,46].

Interestingly, FUS^R^ prevalence in BAHS isolates was markedly higher than in previously reported PJI isolates, where 7 % of *S. aureus* and 56 % of *S. epidermidis* were resistant [19], compared with 33 % and 72 % in the BAHS cohort. FUS^R^ genes were also more prevalent in BAHS isolates than in PJI isolates [19]; for example, 72 % of *S. epidermidis* from BAHS carried *fusB* compared with 40 % in the PJI study. This divergence likely reflects the more frequent topical fusidic acid exposure characteristic of BAHS aftercare, which may preferentially select for strains harbouring *fusB*. Overall, these findings underscore the selective pressure of topical antibiotics, raising concerns about the long-term efficacy of fusidic acid in managing BAHS-associated complications.

Tetracycline resistance genes were also detected across all staphylococcal groups: 11 % in *S. aureus*, 34 % in *S. epidermidis*, and 11 % in other CoNS. All patients received prophylactic treatment with ointment—containing hydrocortisone and oxytetracycline—for up to seven days post-implantation. This routine use of oxytetracycline may contribute to the observed resistance, highlighting the impact of prophylactic topical antibiotics on AMR development. Multidrug resistance remained primarily observed in *S. epidermidis*, consistent with patterns reported in PJI isolates [19].

Beyond acquired AMR from clinical antibiotic use, biofilm formation contributes to increased antibiotic tolerance. MBEC/MIC ratios revealed marked increase in antibiotic tolerance among biofilm-producing strains, with resistance reaching 97 % for fusidic acid, 92 % for clindamycin and 95 % for linezolid under biofilm conditions. Notably, rifampicin resistance of biofilms —a key anti-biofilm agent— was substantially higher in this cohort than in periprosthetic joint infection patients [18,19]. These findings underscore the critical need for biofilm-targeted treatment strategies, including combination therapies and biofilm-disrupting agents. The observed resistance patterns suggest that standard treatments may be insufficient, particularly in patients with adverse local reactions such as pain, debris and inflammation.

### 6.5 Strengths and limitations

Among the study limitations, the small sample size and incomplete follow-up in some patients may have affected the generalizability of the findings. Additionally, multiple isolates from the same patient were analyzed independently, without accounting for potential synergistic or competitive interactions between co-colonizing strains.

Among its strengths, this study is the first to comprehensively characterize *Staphylococcus* spp. isolates from BAHS patients by integrating phenotypic assays, antimicrobial susceptibility testing in both planktonic and biofilm states, and whole-genome sequencing. The combination of clinical, microbiological, and genomic data revealed key associations between clonal lineages, biofilm formation, AMR, and soft-tissue complications. MBEC testing provided valuable insights into biofilm-associated antibiotic tolerance—an often-overlooked factor in implant-related infections. The longitudinal design and multi-site sampling enabled detection of persistent colonization and clonal dissemination over time, offering a detailed view of microbial dynamics around BAHS.

## 7 Conclusion

This study provides the first integrated microbiological and genomic analysis of *Staphylococcus* spp. in BAHS patients, linking clonal lineages, biofilm formation, and antibiotic resistance to clinical outcomes. Persistent colonization and biofilm-associated tolerance highlight the need for early microbial diagnostics and biofilm-targeted therapies. These findings support the development of evidence-based guidelines to improve surgical outcomes and reduce soft-tissue complications in BAHS recipients.

## CRediT authorship contribution statement

**Marsel Ganeyev:** Writing – review & editing, Writing – original draft, Visualization, Validation, Software, Resources, Project administration, Methodology, Investigation, Funding acquisition, Formal analysis, Data curation, Conceptualization. **Liliana Morales-Laverde:** Writing – review & editing, Writing – original draft, Visualization, Software, Methodology, Investigation, Data curation, Conceptualization. **Maria Hoffman:** Project administration, Methodology, Investigation. **Malou Hultcrantz:** Writing – review & editing, Methodology, Investigation, Conceptualization. **Anders Palmquist:** Writing – review & editing, Supervision, Funding acquisition, Data curation, Conceptualization. **Peter Thomsen:** Writing – review & editing, Supervision, Methodology, Funding acquisition, Conceptualization. **Martin L. Johansson:** Writing – review & editing, Validation, Supervision, Project administration, Methodology, Investigation, Funding acquisition, Data curation, Conceptualization. **Margarita Trobos:** Writing – review & editing, Writing – original draft, Validation, Supervision, Software, Project administration, Methodology, Investigation, Funding acquisition, Data curation, Conceptualization.

## Ethics approval

This single-center, sponsor-initiated, prospective, controlled pilot case series (ClinicalTrials.gov, NCT02304692) was approved by the Regional Ethical Review Board of Stockholm (2014/1566-31/2), and conducted per the Declaration of Helsinki and ISO 14155:2011, and all participants provided written informed consent prior to enrolment.

## Funding

This research was funded by the 10.13039/501100011751Swedish Foundation for Strategic Research (SSF; ID20-0091), William Demant Fund (Case no. 20–1587), Oticon Medical AB (Sweden), the Swedish Research Council (2022-00853), the Swedish State under the agreement between the Swedish Government and the country councils, the ALF-agreement (ALFGBG-725641, ALFGBG-1005762), Handlanden Hjalmar Svensson Foundation, Adlerbertska Forskningsstiftelsen, the IngaBritt and Arne Lundberg Foundation, Sylvan Foundation, and the Area of Advance Materials of Chalmers and GU Biomaterials within the Strategic Research Area initiative launched by the Swedish Government.

## Declaration of competing interest

The authors declare the following financial interests/personal relationships which may be considered as potential competing interests:

Marsel Ganeyev is enrolled as an industrial PhD at Oticon Medical AB, fully supported by grants from the Swedish Foundation for Strategic Research (SSF) and William Demant Fund [Case no. 20–1587, https://www.williamdemantfonden.dk/]. Martin L. Johansson is under the employment of Oticon Medical AB (Askim, Sweden). This (commercial affiliation) does not alter the authors' adherence to the journal's policies on sharing data and materials. All other authors declare that the research was conducted in the absence of any commercial or financial relationships that could be construed as a potential conflict of interest.

## Data Availability

Data will be made available on request.
