# Supplementary material for: Staphylococcal persistence and biofilm resistance in bone-anchored hearing systems: Clinical impact
Source: Biofilm. 2025 Dec 13;11:100342. doi: 10.1016/j.bioflm.2025.100342 (PMC12775920; doi:10.1016/j.bioflm.2025.100342)
Supplement: Multimedia component 1 [file mmc1.docx]

# Supplement 1


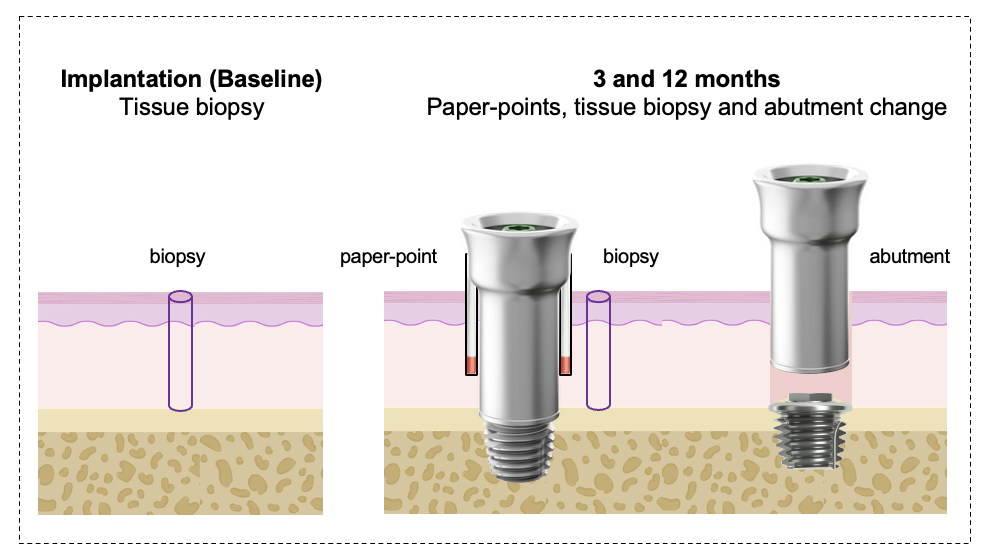


Fig. S1. Microbiological sampling and clinical parameters. Schematics of sampling of tissue with biopsy, peri-abutment fluid with paper-points and retrieval of abutment, at baseline, and at 3 and 12 months according to Trobos *et al*.^1^ Parallel to the microbiological sampling. Additionally, clinical observations, including Holgers score (0-4), debris scores (0-3) and pain scores (0-10), were recorded during each visit.


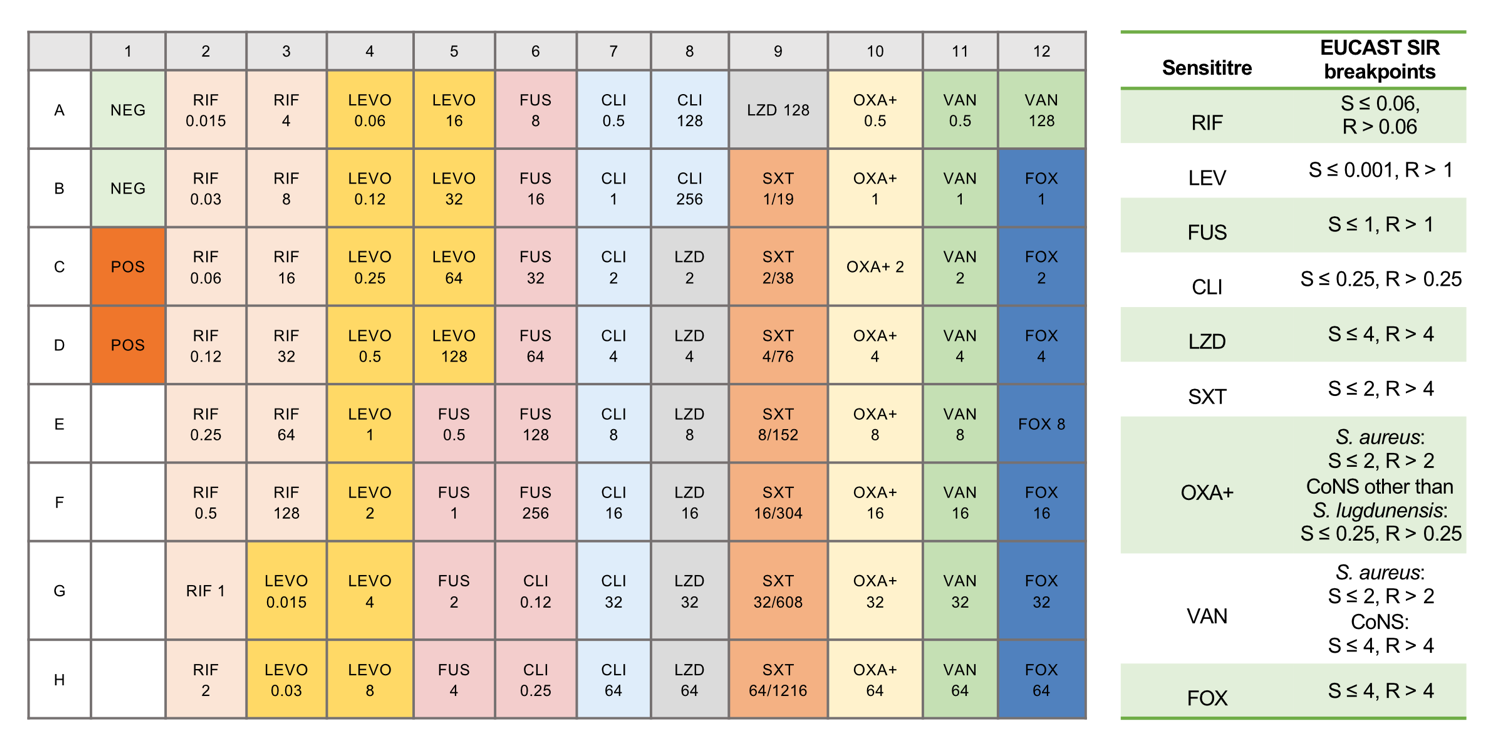


Fig. S2. Antimicrobial agents and concentrations (μg/mL) used in the study. Custom-made antimicrobial susceptibility plate used for the determination of the MIC (minimum inhibitory concentration) and MBEC (minimum biofilm eradication concentration). Four empty wells (1E-H) contained no antimicrobial agent. The following antimicrobial agents were included: Rifampicin (RIF), Levofloxacin (LEV), Fusidic Acid (FUS), Clindamycin (CLI), Linezolid (LZD), Trimethoprim / Sulfamethoxazole (SXT), Oxacillin (OXA+), Vancomycin (VAN), Cefoxitin (FOX). Two wells were used as positive controls (POS) and the other two were used as negative controls (NEG). EUCAST susceptibility breakpoints for SIR determination (S: susceptible, I: intermediate, R: resistant) are displayed on the right.


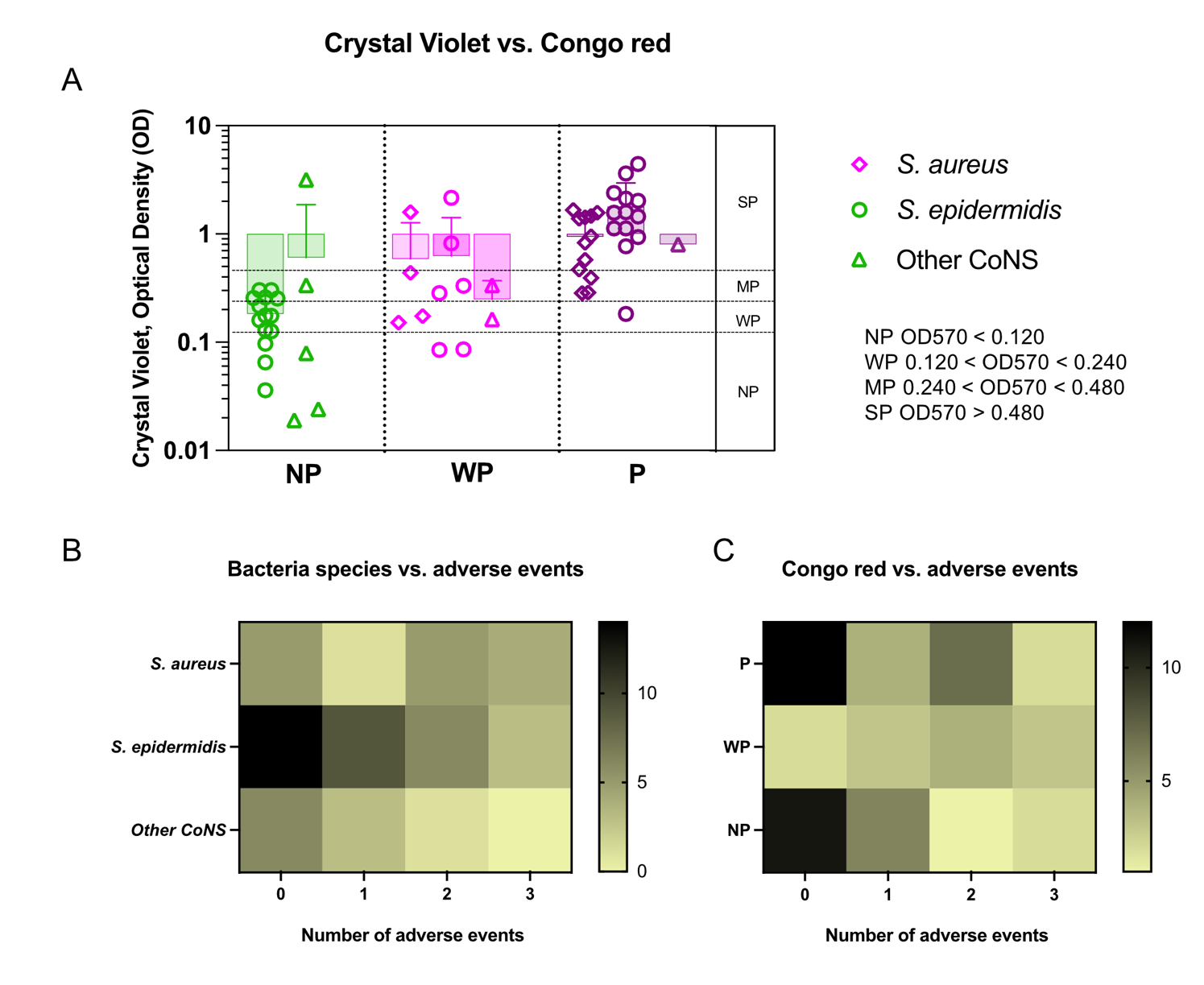


Fig. S3. Biofilm formation, bacterial species, and their association with tissue reactions and pain. (A) Relationship between biofilm formation measured by Crystal Violet assay and slime production assessed using the Congo Red agar assay. The classification of biofilm production follows the criteria established by Baldassarri *et al.*,^2^ where non-producers (NP) have OD_570_ < 0.120, weak producers (WP) OD_570_ =0.120 to 0.240, moderate producers (MP) OD_570_ = 0.240 to 0.480, and strong producers (SP) OD_570_ > 0.480. Different bacterial species are represented by distinct symbols: *S. epidermidis* (circles), other coagulase-negative staphylococci (CoNS, triangles), and *S. aureus* (diamonds). (B) Heatmap showing the association between bacterial species and the number of adverse reactions. (C) Heatmap illustrating the correlation between the Congo Red assay results and the occurrence of skin adverse reactions. Darker shades represent a higher frequency of cases.

**STable 1.** Antimicrobial resistance profiles towards nine agents by MIC (all strains) and MBEC (biofilm slime prducers).

| **Minimum Inhibitory Concentration (MIC)** | All strains  (57) | *S. aureus*  (16) | *S. epidermidis*  (32) | Other CoNS  (9) |
| --- | --- | --- | --- | --- |
| MDR (resistant to ≥3 antimicrobial agents) | 2 (4) | 0 | 2 (6) | 0 |
| Rifampicin (RIF) | 0 | 0 | 0 | 0 |
| Levofloxacin (LEVO) | 0 | 0 | 0 | 0 |
| Fusidic Acid (FUS) | 31 (54) | 5 (31) | 23 (72) | 3 (33) |
| Clindamycin (CLI) | 1 (2) | 0 | 1 (3) | 0 |
| Linezolid (LZD) | 0 | 0 | 0 | 0 |
| Trimethoprim/ Sulfamethoxazole (SXT) | 6 (11) | 0 | 6 (19) | 0 |
| Oxacillin (OXA+) | 2 (4) | 0 | 2 (6) | 0 |
| Vancomycin (VAN) | 0 | 0 | 0 | 0 |
| Cefoxitin (FOX) | 2 (4) | 0 | 2 (6) | 0 |
| **Minimum Biofilm Eradication Concentration (MBEC)** | All strains  (37) | *S. aureus*  (16) | *S. epidermidis*  (18) | Other CoNS  (3) |
| Rifampicin (RIF) | 25 (68) | 12 (75) | 12 (67) | 1 (33) |
| Levofloxacin (LEVO) | 25 (68) | 11 (69) | 13 (72) | 1 (33) |
| Fusidic Acid (FUS) | 36 (97) | 16 (100) | 18 (100) | 2 (67) |
| Clindamycin (CLI) | 34 (92) | 14 (88) | 18 (100) | 2 (67) |
| Linezolid (LZD) | 35 (95) | 16 (100) | 17 (94) | 2 (67) |
| Trimethoprim/ Sulfamethoxazole (SXT) | 29 (78) | 11 (69) | 17 (94) | 1 (33) |
| Oxacillin (OXA+) | 26 (70) | 9 (56) | 16 (89) | 1 (33) |
| Vancomycin (VAN) | 25 (68) | 10 (63) | 14 (78) | 1 (33) |
| Cefoxitin (FOX) | 25 (68) | 9 (56) | 15 (83) | 1 (33) |

*Note: EUCAST breakpoints were used for resistance determination. Abbreviations: MIC: minimum inhibitory concentration; MBEC: minimum biofilm eradication concentration; MDR: multidrug resistance.*

# Supplement 2

## SMethods

### Bacterial typing and carriage of virulence genes: DNA isolation, genome sequencing, and bioinformatic analyses

Genomic DNA was extracted from strains cultured overnight at 37°C on 5% horse blood Columbia agar plates. A single colony was then inoculated into 5 mL of tryptic soy broth (TSB) and incubated at 37°C with shaking at 200 rpm. Following incubation, 1.5 mL of the culture was centrifuged at 16,000 g for 2 minutes, and genomic DNA was extracted from the pellet using the GenElute Bacterial Genomic DNA Kit (Sigma-Aldrich, USA) following the Gram-positive bacteria protocol. This included an initial lysis step with Gram-positive lysis solution supplemented with lysozyme (cat. no. 62971; 2.115 × 10^6^units/mL stock), lysostaphin (200 units/mL), and RNase A to eliminate residual RNA. The DNA was eluted in buffer EB (Qiagen, The Netherlands) and stored at -20°. DNA concentrations and purity were assessed with the Quant-iT™ PicoGreen™ dsDNA Assay (ThermoFisher Scientific, USA), and the DNA was stored at -20°C.

Sequencing libraries were prepared with the Nextera XT kit (Illumina, USA) and sequenced on a MiSeq platform (Illumina, USA), targeting at least 40× coverage. Reads were quality-filtered using TrimGalore (v0.4.0), trimming low-quality ends at a Phred score of 20 and removing reads shorter than 20 bases. Assemblies were annotated with Prokka (v1.14.6), and species confirmation was conducted via multi-locus sequence typing (MLST) using mlst (v2.23.0). Taxonomic classification was performed with Kraken2, and core-genome phylogenetic trees were constructed using a platform at 1928 Diagnostics (Sweden).

Further analyses included plasmid detection, virulence gene identification, and antimicrobial resistance gene screening, all performed using tools from the Centre for Genomic Epidemiology (CGE, Denmark). The *agr* types of *S. aureus* and *S. epidermidis* were identiied by BLASTn searches against contigs, using reference sequences from GenBank: for *S. aureus* (GenBank IDs M21854.1, AF001782.1, AF001783.1, AF288215.1) and *S. epidermidis* (Z49220.1, AF346724.1, AF346725.1). Contigs were converted into BLAST databases, and the highest percentage identity hits were used to assign *agr*types.

# References

1. Trobos M, Johansson ML, Jonhede S, et al. The clinical outcome and microbiological profile of bone-anchored hearing systems (BAHS) with different abutment topographies: a prospective pilot study. *Eur Arch Otorhinolaryngol*. Jun 2018;275(6):1395-1408. doi:10.1007/s00405-018-4946-z

2. Baldassarri L, Bertuccini L, Ammendolia MG, Arciola CR, Montanaro L. Effect of iron limitation on slime production by Staphylococcus aureus. *Eur J Clin Microbiol Infect Dis*. May 2001;20(5):343-5. doi:10.1007/pl00011274
